# Study of the modification of in-depth relative permeability modifier (RPM) by graft zeolite copolymers for conformance control

**DOI:** 10.1039/d5ra00177c

**Published:** 2025-02-27

**Authors:** Sofiah Atirah Raya, Ismail Mohd Saaid, Dzeti Farhah Mohshim, Ahmad Amirhilmi A Razak

**Affiliations:** a Petroleum Engineering Department, Universiti Teknologi PETRONAS 32610 Bandar Seri Iskandar Perak Malaysia sofiah_22009555@utp.edu.my; b PETRONAS Research Sdn Bhd, Petronas Research & Scientitic Jln Ayer Hitam 43000 Bandar Baru Bangi Selangor Malaysia

## Abstract

The most used technique for controlling high water production in mature oil fields is conformance control agent with high swelling behavior and stability. However, conformance control destabilization often occurs from particle aggregation leading to particle migration (creaming or sedimentation). Coalescence or cluster formation at an early stage of conformance control is crucial for increasing thermal stability and enhancing formulations. The objective of this study was to characterize the colloidal dispersion stability of grafted zeolite copolymers to assess its use as potential conformance control in carbonate reservoirs. In this study, grafting form of bentonite, acrylamide (AM), 2-acrylamino-2-methylpropanesulfonic acid (AMPS), zeolites with anionic surfactants (Sodium Dodecyl Sulphate, SDS) and nonionic surfactants (Span 80 and Tween 80) were prepared. Stability during multiple light scattering analysis of grafted bentonite with and without modification were observed and compared through Turbiscan Classic MA 2000. The results revealed that the multiple light scattering analysis and colloidal dispersion stability all reflect the stability system of the grafted bentonite. Indeed, the multiple light scattering analysis was able to be used to derive the stability for graft zeolite copolymers and showed good agreement with the results from turbidity. The addition of zeolites combined with Span 80 and Tween 80 was observed to enhance stability, maintaining a 1% transmission value over 60 minutes, which is classified as having “good” characteristics for conformance control.

## Abstract 2

In this energy region, bands from grafted bentonite have lower values of transmittance (%) and downward spikes and thus a higher absorption peak than pure bentonite. This spectrum demonstrates that polymers are appropriately located on the surface of bentonite particles.

## Graphical abstract

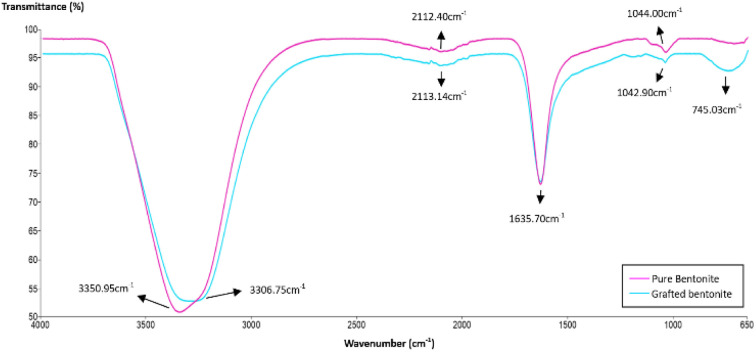

## Introduction

As the demand for oil and gas continues to grow, extending the lifespan of mature hydrocarbon reservoirs remains significant for the coming decades. While the demand for hydrocarbons remains critical, one of the main challenges encountered during extraction efforts in oilfield operation is excessive water production. These challenges often lead to reduced recovery efficiency including corrosion, fines migration, fluid pressure load and increased operational costs, underscoring the need for effective solutions.^1^ Conformance control has been introduced as a technology that employs chemical or mechanical methods to reduce or block water or gas production caused by wellbores, high-permeability zones, channels, or fractures in reservoirs.^2,3^

Different methods have been utilized as conformance control for treating high water production in wells, such as gel treatments, polymer flooding, relative permeability modifier (RPM) and alkaline-surfactant-polymer (ASP) treatment.^4–7^ Among these methods, RPM is one of the most widely used resolutions in enhancing fluid recovery in subsurface operations, particularly in petroleum reservoirs and groundwater systems. It aims to improve sweep efficiency by sealing high-permeability zones in porous media and modifying fluid flow properties.^8–10^

In carbonate reservoirs, these challenges are further exacerbated by their inherent heterogeneity and high permeability contrasts, making advanced conformance control strategies even more crucial. Carbonate reservoirs pose unique challenges due to their complex pore structures and chemical reactivity, which can affect the behavior and stability of colloidal dispersions. The RPM efficiency is demonstrated by the colloidal stability under reservoir conditions, which is influenced by their intermolecular interactions.^11^ Instability issues, such as sedimentation, aggregation, or phase separation, can compromise their effectiveness, making it essential to study and optimize their behavior under operational conditions.

Zeta potential and ageing test are the ways to obtain the stability behavior of a dispersion. However, zeta potential measurements are often unsuitable for complex industrial formulations as they fail to provide information on the flocculation behavior of the particles in the continuous phase. Ageing tests, on the other hand, rely on visual observation to detect physical stability, which is particularly unreliable especially for concentrated liquid dispersions.^12^ Microscopy, spectroscopy, and multiple light scattering have been introduced for a more precise analytical observation of the stability of colloidal dispersions in liquid suspensions.^13^

Numerous studies have demonstrated the potential of nanoparticles for use in enhanced oil recovery applications.^14,15^ At present, bentonite particles have received extensive attention due to their highly swelled properties. Ahmed *et al.*^16,17^ have investigated the impact of co-polymer grafted bentonite for conformance control in heterogenous reservoir. They successfully modified bentonite clay and poly (NIPAM-*co*-AA). The results showed a significant reduction in permeability, with up to 74% of the initial permeability being decreased after injecting the grafted bentonite. Bentonite exhibits varying swelling properties in brines, forming aggregates and settling rapidly due to increased electrostatic forces.^18^ However, the swelling properties can be regulated and inhibited by modifying the interlayer exchangeable cations.^19,20^ It has also been reported that bentonite modified by ion exchange ion exchange at 100% of its cation exchange capacity (CEC) exhibits enhanced organophilic properties, improved thermal stability, and strong potential for application in conformance control.^21^

In the context of this study, zeolites nanoparticles are of particular interest for their potential to improve conformance control. Zeolites possess a well-defined microporous framework that introduces functional groups to increase the number of active sites, while their hydrophilic or hydrophobic nature can be tailored through chemical modification.^22,23^ An *et al.*^24^ have shown experimentally that zeolite nanoparticles have an influence on oil viscosity, interfacial tension and wettability alteration. In their work, the oil recovery was improved and reached 70% of the original oil in place (OOIP). Wang *et al.*^25^ studied the influence of zeolite in interpenetrating polymer network (IPN). The IPN plugging agent demonstrated a stability level 3.8 times higher than that of the conventional polyacrylamide (PAM) plugging agent. Zeolite crystallization has been reported successfully achieved through modification with crosslinked polyacrylamide hydrogels, which provide a supportive network for zeolite growth and facilitate the formation of their structures.^26^

At present, there a good research results about the best form of nanoparticle copolymers with surfactants as conformance control.^27–29^ Nonionic surfactants has advantage to adhere the surface through steric interactions when in contact with clay-water suspensions, because they lack charged groups and interact with surfaces through hydrogen bonding or hydrophobic forces. By preventing close contact, steric interactions can enhance colloidal stability of the RPM.^30,31^ It has high potential on RPM formulation on grafted bentonite modified with zeolites and non-ionic surfactants. However, little work has been carried out and comprehensively presented the colloidal dispersion stability of the formulation.

Despite advances in colloidal technology for conformance control, there is a lack of studies on the stability of colloidal dispersions, particularly in conditions that simulate carbonate reservoirs. The formulation components of RPM such as the salinity, monomers, zeolite and non-ionic surfactants concentration might affect the performance of conformance control. Compatibility and colloidal dispersion stability tests were conducted to explore further and confirm the potential of grafted bentonite with and without modifications as in-depth RPM in carbonate reservoir. This study aims to address this gap by using Turbiscan to investigate the stability of zeolite-surfactant dispersions. Firstly, aqueous grafted bentonite dispersions in different monomers concentration and salinities were studied. Secondly, the zeolites and three ionic surfactants, sodium dodecyl sulfate (SDS), Span 80 and Tween 80 are used in various concentrations. The findings will provide valuable insights into optimizing colloidal formulations for enhanced conformance control performance in carbonate reservoirs.

## Materials and method

The bentonite used in this study is a type of montmorillonite clay. Grafted bentonite (GB) solution was prepared by grafting the bentonite colloids with acrylamide (AM), 2-acrylamino-2-methylpropanesulfonic acid (AMPS), 3-[dimethyl-(2-methyl-prop-2-enoyloxy)ethyl-azaniumyl] propane-1-sulfonate (DMAPS), ammonium persulfate (APS), zeolites, sodium dodecyl sulfate (SDS), sorbitan monooleate (Span 80), and polysorbate monooleate (Tween 80). Zeolite nanoparticles used in this study is nano-beta zeolite which is known to be a versatile alternative adsorbent for Volatile Organic Compounds (VOCs) and separation of aromatics from alkanes and mixtures of an alkene. The zeolite also has a purity of >99% and specific gravity of 2.0 g cc^−1^. The dispersion velocity of 0.2 wt% grafted bentonite is 1.35 cP at 24 °C. Sodium chloride (NaCl) was used to prepare brine in this study. The chemicals were purchased from Sigma-Aldrich (M) Sdn Bhd, Malaysia.

### Graft copolymerization of conformance control

Hydrophilic sodium bentonite was used to formulate the conformance control agent. For 2% stock solution preparation in 2.5% NaCl, 2 g nano powder bentonite was added into 1 L distilled water (DI) and stirred at 400 rpm for 6 hours. DMAPS was added and stirred at 400 rpm for another 6 hours. Other monomers are copolymerized with PAM to enhance the thermal stability of the base PAM copolymer. Adding these groups reduces the tendency of hydrolysis.^32^ Grafting was performed under a nitrogen atmosphere through free radical polymerization of AM and AMPS onto the surface of modified DMAPS-bentonite particles in an aqueous solution. A ratio of 42% : 58% – AM : AMPS was added into DMAPS-bentonite dispersion (ratio 1 : 3) and continued stirred at 80 rpm for 2 hours. Various ratios of AM : AMPS to the monomer mixture, ranging from 42% : 58%, 50% : 50%, and 58% : 42%, were used for the polymerization reaction. Then, APS was added and stirred at 80 rpm for 2 hours. The solution was vacuumed for a minimum of 30 minutes, and then nitrogen equivalent to atmospheric pressure was used to break the vacuum. Then, the solution was heated to 70 °C and stirred at 80 rpm for 2 hours. Finally, the polymer-grafted bentonite was subsequently mixed with salinities 0, 2.5, and 3.5 wt% NaCl. The same procedure was repeated with zeolites of varying concentration (0.005%, 0.015% and 0.01%), anionic surfactant, SDS (0.001 M, 0.01 M, 0.1 M) and nonionic surfactants, Span 80 and Tween 80 (0.1%).

### Functionalization of carbonate with grafted bentonite

A compatibility test of conformance control was conducted to understand the functionalization of the grafted bentonite with carbonate rocks. The bentonite sample was tested using ASTM-D5890 swell index procedure (free swell test). A dried and finely ground bentonite clay sample weighing 2 g was added to a 100 ml graduated cylinder incrementally, with each increment being 0.1 g. Then, the sample was left for 24 hours. The time for the bentonite to settle down was recorded. Then, a batch gravimetric was conducted to determine the adsorption capacity of the carbonate rock particles. Subsequently, each GB solution was immersed in approximately 60 grams of carbonate rock particles in a plastic beaker for 48 hours. The rock particles were thoroughly rinsed with deionized water and dried in an oven for 12 hours at 60 °C. Then, the weight difference of the dried particles before and after the adsorption process was measured. The GB solution obtained after the adsorption experiments was analyzed using FTIR (Fourier Transform Infrared) and compared with the original solutions before carbonate adsorption.

### Characterization of grafted bentonite

After formulating the conformance control agents, experimental techniques were employed to investigate their characteristics. These characterizations aimed to assess the structure of grafted polymerized bentonite and determine various properties, including morphology, colloidal dispersion stability, particle size distribution, and rheology. FTIR analysis was employed to compare the structural changes of pure bentonite, and graft polymerized bentonite. Then, the colloidal dispersion stability was determined using light transmission and backscattering by Turbiscan Classic (MA2000).

#### Fourier-transform infrared spectroscopy (FTIR) analysis

FTIR analysis was conducted using a Thermo Scientific Nicolet iS10 FT-IR Spectrometer to analyze the pure bentonite and grafted polymerized bentonite. The analysis was performed at a resolution of 4 cm^−1^ with 32 scans, covering the 4000–400 cm^−1^ range. The samples were heated at 100 °C to remove excess water. The dispersion of the grafted bentonite was vacuum dried using a desiccator and then carefully ground to obtain a finely powdered form before analysis. Next, for the adsorption capacity test, the infrared (IR) spectra were attained within the wavelength range of 4000 to 800 cm^−1^ by averaging 32 scans at a resolution of 2 cm^−1^.

#### Scanning electron microscope (SEM)

Core samples were sectioned into small pellets to facilitate imaging analysis using scanning electron microscopy (SEM). A Evo LS15 VPSEM model SEM was employed for imaging, which utilizes electron beams instead of light to generate high-resolution images of the samples. For sample preparation, a diluted aliquot of the dispersions was carefully dropped onto a glass chip and left to dry under ambient conditions before analysis. SEM images of both pure and grafted bentonite were captured at a magnification of ×500 and ×3000 to evaluate surface morphology and structural modifications.

#### Particle size analysis (PSA)

Particle dispersions were obtained using ultrasonication, by dispersing a known weight of bentonite, zeolite nanoparticles powder in the water. Deionized water was employed to prepare all particle suspensions. They will either deposit or separate into individual dispersed phases when the aqueous phases forming nonionic surfactants were unstable. The size and stability of the aqueous phase were observed using a Malvern Zetasizer Nano ZSP and repeated three times.

#### Multiple light scattering analysis

The dispersion stability of grafted bentonite with and without modification was studied by Turbiscan Classic (MA 2000) based on the principle of dynamic light scattering. The Turbiscan head consists of a pulsed near-infrared light source (*λ* = 850 nm) and two synchronized detectors: a transmission detector that captures light passing through the sample (at 0° relative to the incident beam) and a backscattering detector that captures light scattered backward by the sample (at 135° relative to the incident beam). The conformance control systems in ratio of 42% : 58%, 50% : 50%, and 58% : 42% different polymers were prepared in deionized water. The solution of 7 ml was added to the test tube, which was placed in the stability analyzer. The parameter termed as delta transmission (Δ*T*), and backscattering (ΔBS) intensities were used to indicate the stability of the dispersed system. The tests were repeated for grafted bentonite in varying salinities range from 0 to 3.5 wt% NaCl. The samples were stirred well after mixing with brine. Then, grafted bentonite in the most stable salinity was modified with zeolites and coated with anionic surfactant (SDS) and nonionic surfactant (Span 80 and Tween 80). Next, turbidity tests were conducted on the same samples to explore their colloidal dispersion stability and support the results obtained from multiple light scattering analysis.

#### Rheology

Viscosity measurements of the dispersion were carried out using the Anton Paar Physica MCR-302. The double gap geometry DG-26.7 was selected for all samples, assuming a viscosity below 0.01 Pa s. For each sample, 0.2 wt% bentonite was dispersed in deionized water followed by 24 hours of mixing before rheological measurements. An adsorption time of 24 hours was employed to ensure complete bentonite hydration. The viscosity of the GB and modified GB samples was measured at temperatures of 60–120 °C.

## Results and discussion

In this section, the characterization results of carbonate samples are outlined. Compatibility tests were performed on carbonate core and formulated conformance control agent based on the procedures in the previous chapter.

### Compatibility between grafted bentonite and carbonate rocks

According to the differential weights of cores before and after aging, compatibility between the grafted bentonite and carbonate rock can be determined. The results are tabulated in Table 1. It was observed that the recovery of cuttings exceeded 99%, with no apparent alterations in color or shape following aging, indicating an outstanding compatibility.

**Table 1:** Compatibility between grafted bentonite and carbonate cores

Core	Weight of core (g)		Recovery percent (%)
	Before being contaminated	After being contaminated	
1 (2.5 wt% GB)	20.29	20.18	99.5
2 (3.5 wt% GB)	20.75	20.61	99.3
Average			99.4

### FTIR analysis

**Fig. 1:**
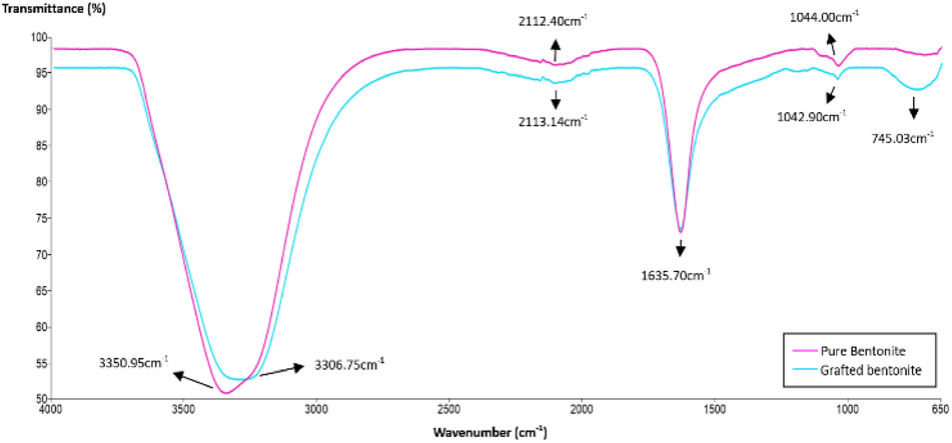
FTIR spectra of pure bentonite and grafted bentonite.

FTIR spectra were determined using a Thermo Nicolet FTIR spectrometer. The objective of this analysis is to identify the functional groups within grafted bentonite and carbonates. Transmittance bands of bentonite before and after modification ranging from approximately 650 to 4000 cm^−1^ were analyzed and shown in Fig. 1. As it is illustrated in the spectra of pure bentonite, a sharp O–H stretching bending of bentonite structure and surrounded water was observed at 3350 cm^−1^.^33^ At the frequency of 3306 cm^−1^ in the spectra of grafted bentonite, a stretching and bending of N–H amine was observed due to the presence of acrylamide units within the polymer molecules.^34^ The adsorption band at 2112.40 cm^−1^ and 2113.14 cm^−1^ extend the vibrations of symmetric and anti-symmetric carbon-hydrogen (C–H) bond of aliphatic alkanes. The peak at 1635.70 cm^−1^ in both spectra of pure bentonite and grafted bentonite corresponds to the linear carbon–oxygen (C–O) stretching and CH_3_, which exist in the polymer chain.^35^ The bonds belong to the polymers and the bentonite clay used. Peaks corresponding to silicon–oxygen (Si–O) bonds were identified from the absorption spectra at 1042.90 cm^−1^ and 1044.00 cm^−1^. The peak at 745 cm^−1^ is associated with the quartz admixture in the sample. In this energy region, bands from grafted bentonite have lower values of transmittance (%) and downward spikes and thus a higher absorption peak than pure bentonite. This spectrum demonstrates that polymers are appropriately located on the surface of bentonite particles.

**Fig. 2:**
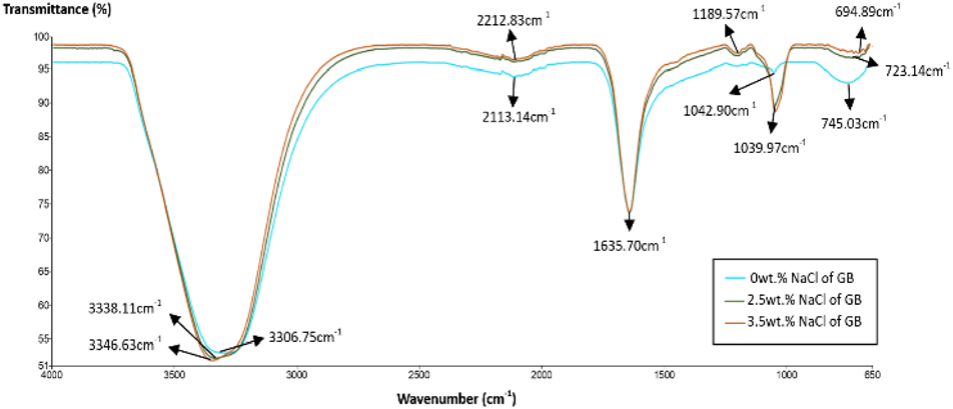
FTIR spectra of 0 wt%, 2.5 wt% and 3.5 wt% NaCl grafted bentonite.

The FTIR spectra of 0, 2.5 and 3.5 wt% NaCl grafted bentonite respectively were plotted in Fig. 2. As shown in the spectra, the band in the region of 1189.57–1039.97 cm^−1^ was associated with stretching vibration of Si–O group. A sharp band at 1039.97 cm^−1^ for 2.5 and 3.5 wt% NaCl grafted bentonite is attributed to perpendicular Si–O stretching. The significant change in Si–O stretching band suggests that there is a stronger molecule interaction between polymer molecules and siloxane (Si–O) surface in grafted bentonite.^36,37^

**Fig. 3:**
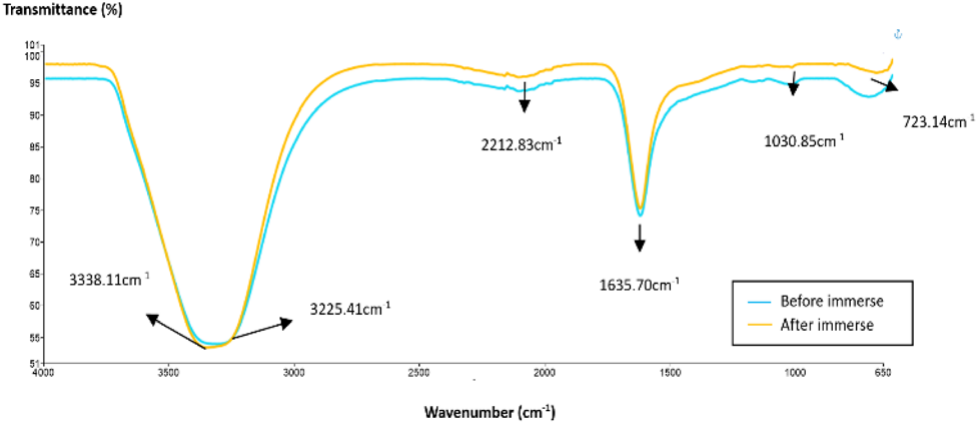
FTIR spectra of grafted bentonite before and after immersing in high salinity carbonates.

Considering the conditions of the carbonate reservoir, the grafted bentonite was tested under high salinity in carbonate environments. A comparison was made between grafted bentonite before and after immersion in 65 g per L NaCl carbonate rock in Fig. 3. As illustrated in the figure, the vibration band at 1030.85 cm^−1^ does not coincide with Si–O band in grafted bentonite. The detection shows the most likely same mineralogy between grafted bentonite and grafted bentonite when immersed in carbonates with increase salinity (65 g L^−1^). The low bending vibration at lower wavenumbers suggests that silica content in carbonate rocks is very low.

**Fig. 4:**
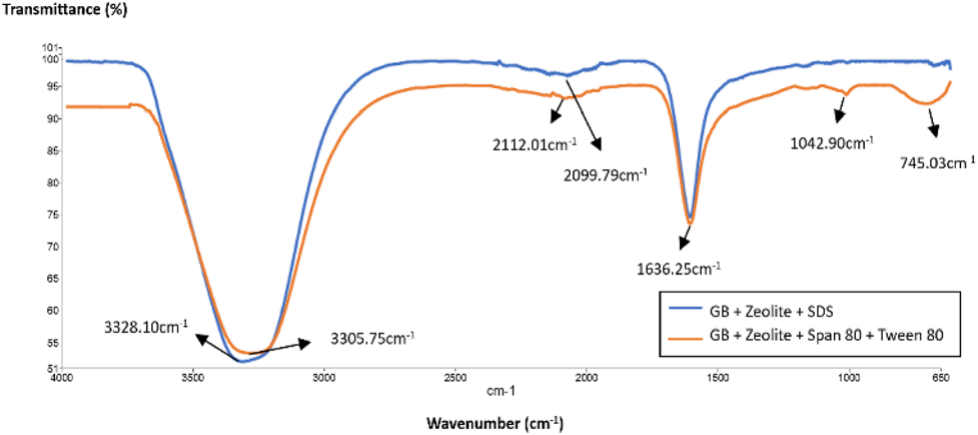
FTIR spectra of modified grafted bentonite with SDS surfactant, Span 80 and Tween 80.

Fig. 4 illustrates the impact of SDS surfactant, Span 80, and Tween 80 in deionized water on the FTIR analysis of zeolite-modified grafted bentonite (GB). Compared to the original GB (Fig. 1), the modified GB, zeolite, and the Span 80–Tween 80 mixture band are stronger. The reduced transmittance values correspond to downward spikes, indicating higher absorption peaks. The results suggest that the functional groups in the sample remain unchanged, indicating that no new bonds were formed or broken. The transmittance of SDS-modified GB exhibits peaks at 3328.10 cm^−1^ and 1636.25 cm^−1^, corresponding to O–H stretching, C–O stretching, and CH_3_ vibrations within the bentonite structure. A slight bending is observed at 2099.79 cm^−1^, representing the symmetric and anti-symmetric vibrations of C–H bonds in aliphatic alkanes. Notably, the Si–O stretching band is absent in SDS-modified GB. These findings suggest that SDS surfactant restricts deformations in bentonite particles due to intercalation.

### Scanning electron microscope (SEM)

**Fig. 5:**
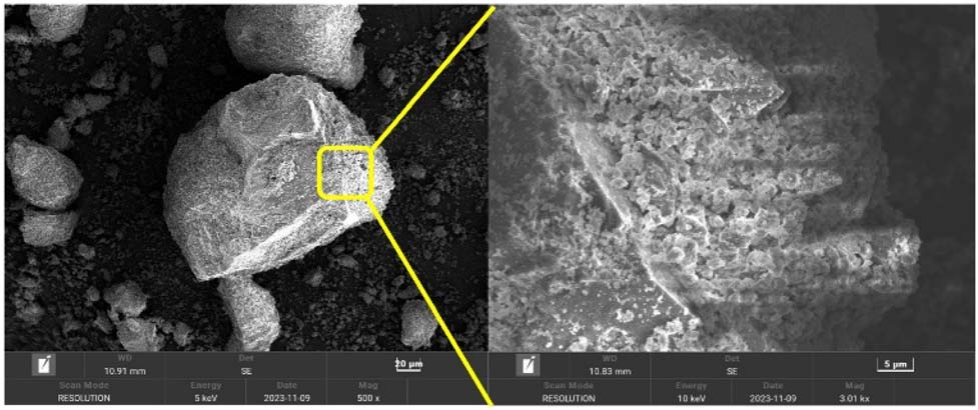
SEM images of carbonate rocks.

The crystalline amorphous of the carbonates was further observed by the scanning electron microscope (SEM). The microstructures of carbonate rocks are observable as represented in Fig. 5. Carbonate rocks appeared to be a single crystal and exhibited the characteristic euhedral to subhedral morphology, with sizes ranging from 1.0 to 3.5 μm.^38^

**Fig. 6:**
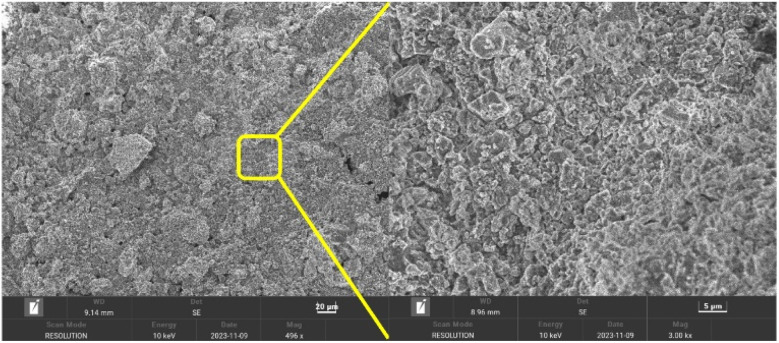
SEM images of the grafted bentonite in carbonate rocks.

Then, the morphology of bentonite after grafting was observed in the carbonate rocks. As shown in Fig. 6, the flake-like structure revealed a large amount of polymerization at the interface of bentonite and carbonates. This implies that the polymer chains attached to the bentonite particles improve their anti-agglomeration properties.^39^ In addition, the grafted bentonite demonstrated a good distribution form in the carbonates, as it displayed the size of ±1.2 μm. The SEM analysis distinctly revealed the significant difference in crystal morphology of the grafted bentonite, which exhibited effective polymerization.

### Multiple light scattering (Turbiscan)

Turbiscan technology is well-known to provide information on dispersion stability of a formulation, which is a significant index for GB system. Effective dispersion stability helps maintain the concentration of GB during preparation, injection, and migration, which improves the plugging ability of the dispersed GB system and reduces economic costs. This part discusses the stability analysis of bentonite dispersions in deionized water and saline water of 2.5 and 3.5 wt% NaCl using Turbiscan. The dispersions studied are pure bentonite, bentonite with monomer, bentonite coated with zeolites, anionic surfactant (SDS) and nonionic surfactants (Span 80 and Tween 80).

**Fig. 7:**
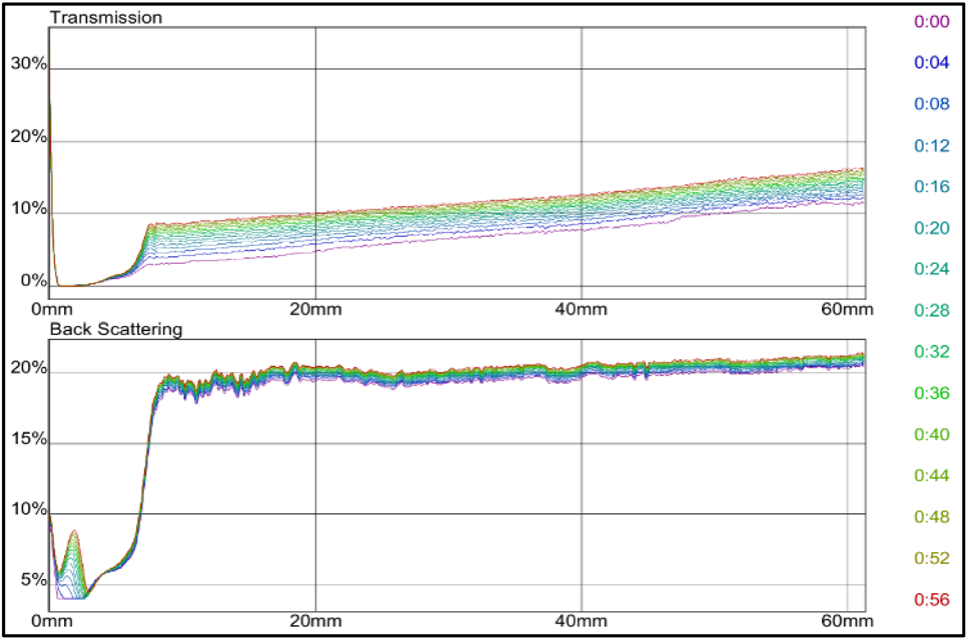
Transmission and backscattering profiles of pure bentonite.

Light transmission, scattering, or reflection occurs when a beam of light interacts with a medium involving dispersed particles. Fig. 7 shows the dependence of delta transmission (Δ*T*), and backscattering (ΔBS) intensities of pure bentonite dispersion obtained by Turbiscan (MA 2000) on sample height and time, where the larger the transmission value, the more unstable the system. As time increased, the light transmittance increased, signifying a linear decline in the concentration of colloidal particles. If the bentonite particles are significantly larger than the wavelength of the incident light, light reflection dominates, making the medium opaque. As the particle size decreases to below the light's wavelength, scattering occurs, causing the medium to appear transparent. When particles are much smaller than the wavelength, light passes through, and the solution becomes fully transparent.^40^

**Fig. 8:**
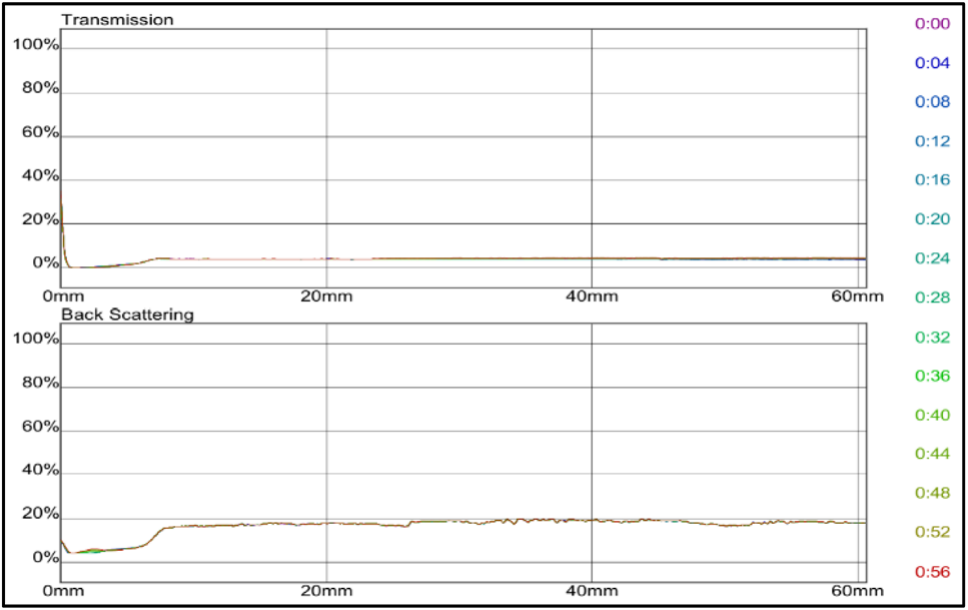
Transmission and backscattering profiles of bentonite polymerized with AM 42% : 58% AMPS.

**Fig. 9:**
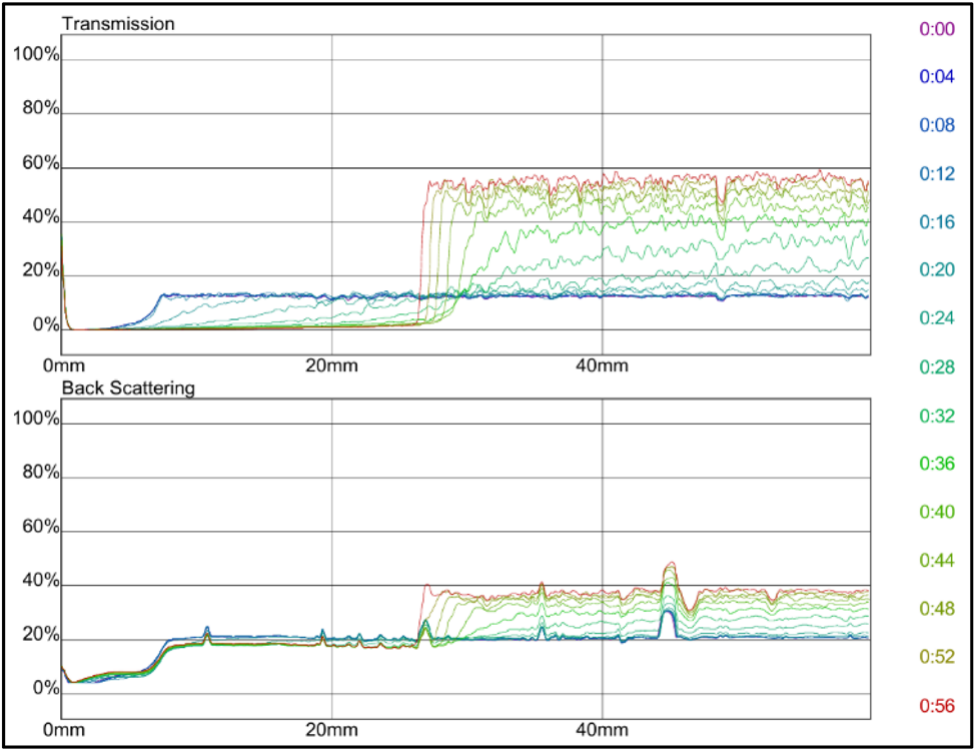
Transmission and backscattering profiles of bentonite polymerized with AM 50% : 50% AMPS.

**Fig. 10:**
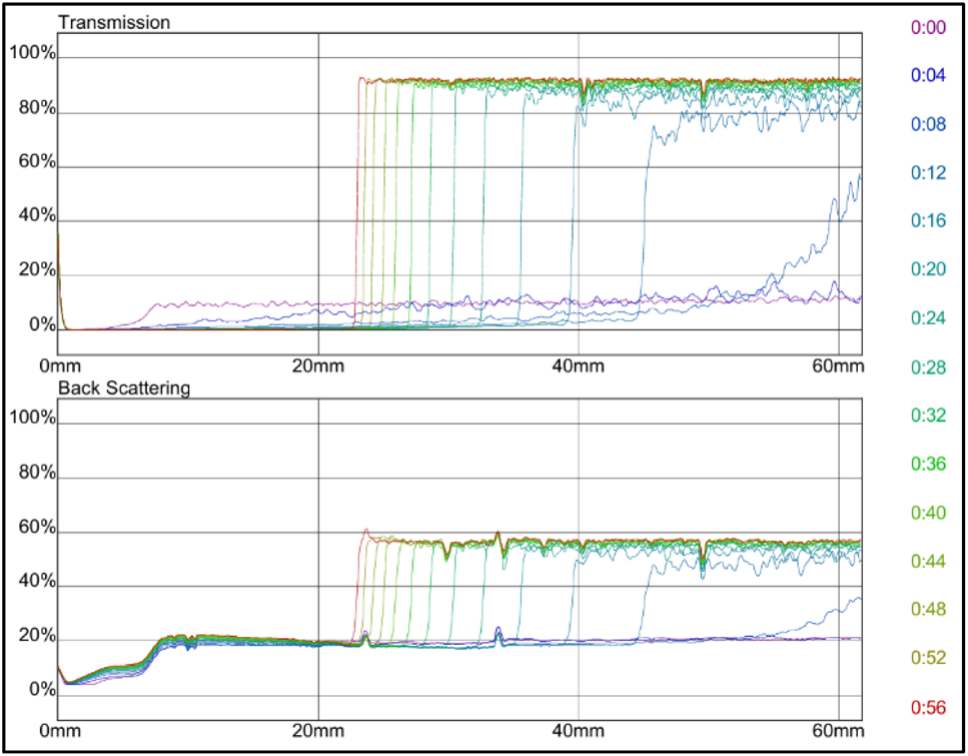
Transmission and backscattering profiles of bentonite polymerized with AM 58% : 42% AMPS.

Furthermore, the stability is further improved by adding combined polymer acrylamide, AM and 2-acrylamino-2-methylpropanesulfonic acid, AMPS, where both polymers have good dispersion stability. The effect of monomers AM and AMPS was investigated. The monomer concentration varied in ratios of AM : AMPS between 42% : 58%, 50% : 50%, and 58% : 42% as displayed in Fig. 8–10 respectively. Among the three solutions, the dispersion stability of polymer AM 42% : 58% AMPS was the strongest. The transmission intensities value tended to be stable at 4% in 60 min, and the stability level of conformance control system was in good condition. The result of this sample is consistent with the research conducted by A. A. Roslan *et al.*^41^ They found out that conformance control involving monomers with ionizable functional groups at increased concentration had better swelling performance and was significant in improving dispersion stability. The polymer can enhance the viscosity of the aqueous phase, thereby increasing the fluid's internal friction.^5^

Meanwhile, the dispersion stability of grafted bentonite polymerized by AM 50% : 50% AMPS was poorer, and the conformance control system was unstable after 32 min as the transmission intensities showed up to 58%. Transmittance intensities were time dependent at time below half an hour before reaching a plateau. For sample AM 58% : 42% AMPS, the colloidal dispersion stability was poorer than AM 50% : 50% AMPS. At 12 min of the test, the sample tended to be unstable and reach transmission intensities up to 97%. In general, the higher the transparency of the medium, the more unstable the medium, the greater the intensity of the transmitted light.^42^ A greater variation of the transmission profiles was detected as the concentration of monomers increased. The results are taken to be higher monomer content showed less exfoliation with less stability, leading to enhanced scattering intensity. In polymeric materials, the monomer-to-precursor ratio influences the extent of crosslinking and internal structure. Increased crosslinking may produce more compact particles with higher scattering efficiency. Pure bentonite (Fig. 7) showed better stability than AM 50% : 50% AMPS (Fig. 9) and AM 58% : 42% AMPS (Fig. 10). Pure bentonite is naturally hydrophilic due to its negatively charged layers, which attract water molecules and create a stable colloidal dispersion. The addition of monomers enhanced water compatibility and steric stabilization. However, an excess of hydrophilic groups can lead to excessive swelling, potentially compromising mechanical strength.^43^

**Fig. 11:**
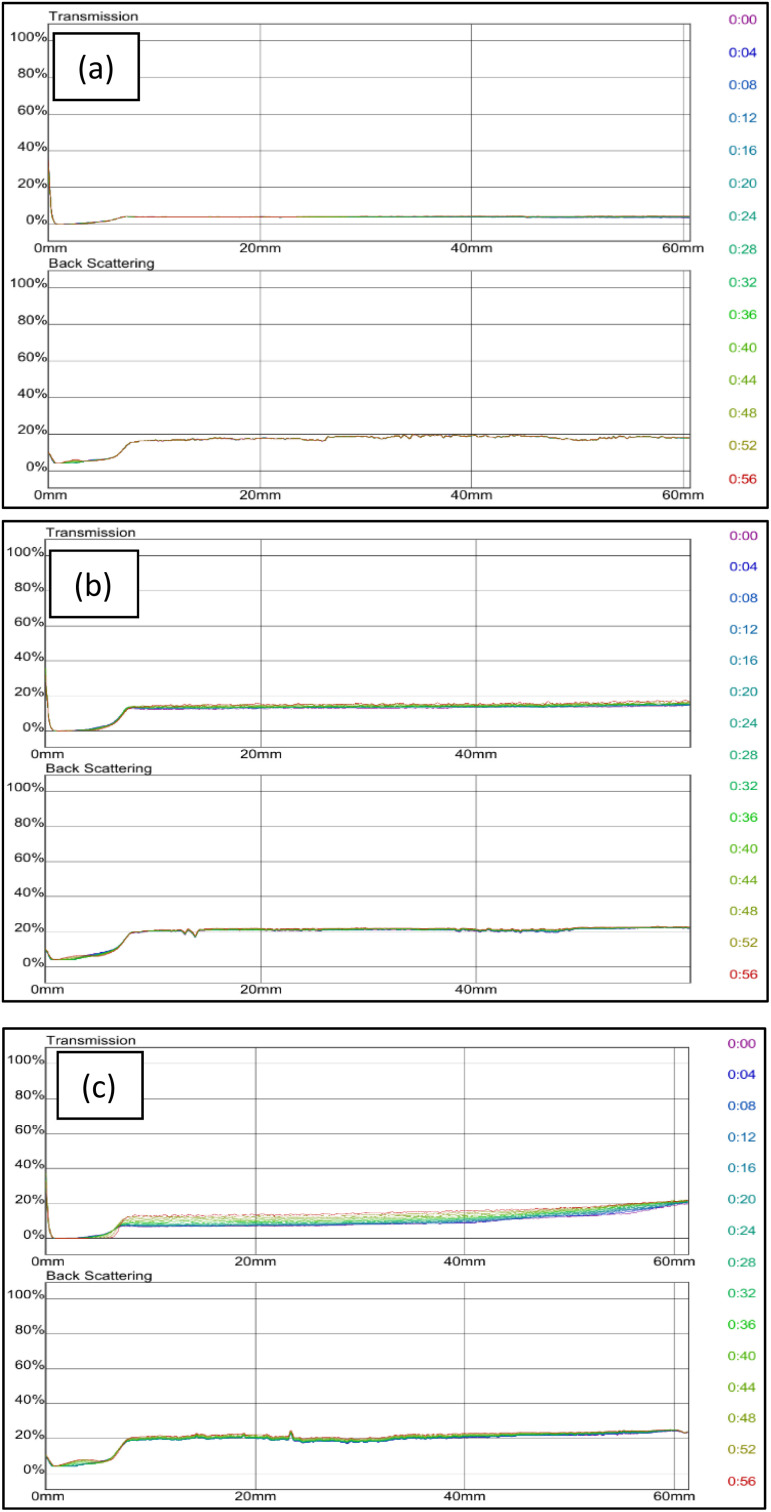
Transmission and backscattering profiles at (a) 0 wt%, (b) 2.5 wt% and (c) 3.5 wt% NaCl of grafted bentonite.

Since grafted bentonite with polymer ratio of 42% : 58% of AM is the most stable compared to the two other polymers concentrations, it was chosen to be the optimum grafted bentonite to be modified in saline water, zeolites and surfactants. Fig. 11 shows the dependence of delta transmission (Δ*T*), and backscattering (ΔBS) intensities of grafted bentonite dispersion in 0, 2.5 and 3.5 wt% NaCl obtained by Turbiscan (MA 2000) on sample height and time. In Fig. 11(a), the transmission intensities value of 0 wt% NaCl grafted bentonite tended to be stable at 4% in 60 min, which is the least compared to the other two grafted bentonite samples. In Fig. 11(b), the transmission intensities value of 2.5 wt% NaCl grafted bentonite tended to be stable in the range of 13% to 17% after 60 min, which is less than 0 wt% NaCl grafted bentonite. In Fig. 11(c), the transmission intensities value of 3.5 wt% NaCl grafted bentonite tended to be stable in the range of 7% to 20% after 60 min. Grafted bentonite from 0 wt% NaCl shows a more significant impact on transmission and backscattering profiles, with grafted bentonite with 2.5 wt% NaCl and 3.5 wt% NaCl shows higher transmission and backscattering profiles. The settling rate and sediment volume decreased with decreasing concentration of NaCl, due to greater double layer compression resulting from higher ionic strength. In addition, NaCl reduces the dispersibility and stability of grafted bentonite by increasing the electrostatic attraction between the polymer series.^40^ The findings were proven with the suspended particles separated at a fast rate observed in the presence of NaCl.

**Fig. 12:**
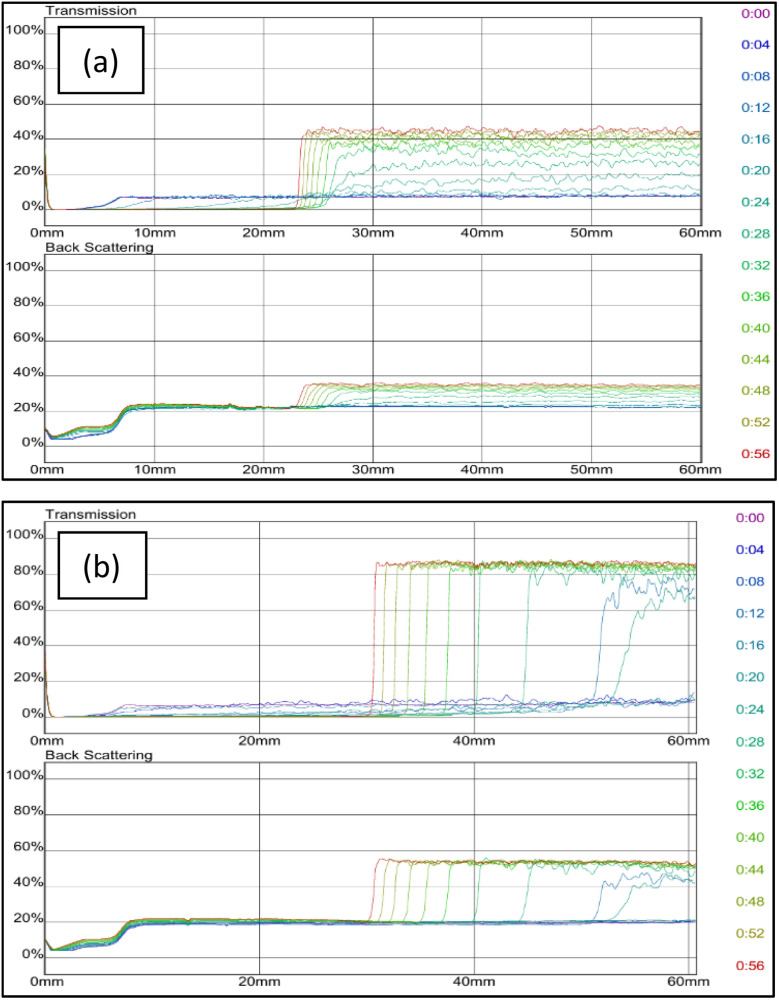
Transmission and backscattering profiles of grafted bentonite modified with (a) zeolites and (b) zeolites with SDS.

Consequently, for the subsequent experimental procedures, the chosen brine concentration was 0 wt%. Grafted bentonite was modified with zeolites coated with anionic surfactant (SDS) and nonionic surfactant (Span 80 and Tween 80). Fig. 12 shows the dependence of delta transmission (Δ*T*), and backscattering (ΔBS) intensities of grafted bentonite dispersion with zeolites and addition of SDS surfactants obtained by Turbiscan (MA 2000) on sample height and time. For graft copolymer zeolites (a), in the middle area within 24 mm, the transmitted light intensity reached a plateau and with the increase of time, and the graph shows that the transmitted light intensity of the upper liquid surface is quite large at around 45%. This is believed to occur due to the suspended particles undergo coagulation–flocculation because of the addition of solid zeolite nanoparticle flocculant. Then, the formation of a clarified zone occurs once the particles settle.

By adding SDS surfactants in the graft copolymer zeolites (b), for 16 minutes, the transmittance at the bottom of the test bottle is approximately 10%. In the middle section transmittance exceeds 60%, while in the top section, it is generally above 70%, with a maximum value of 94%. This is thought to be the addition of SDS can alter the surface potential and influence the electrostatic interactions between the suspended particles. This highlights the reduction of electrostatic repulsion between the particles and dispersion stability.^44^ During early scanning of both samples (a) and (b), slight fluctuations in transmittance were observed between 0 mm and 7 mm which could be explained by the settling down of the suspended particles at the bottom of the bottle.

**Fig. 13:**
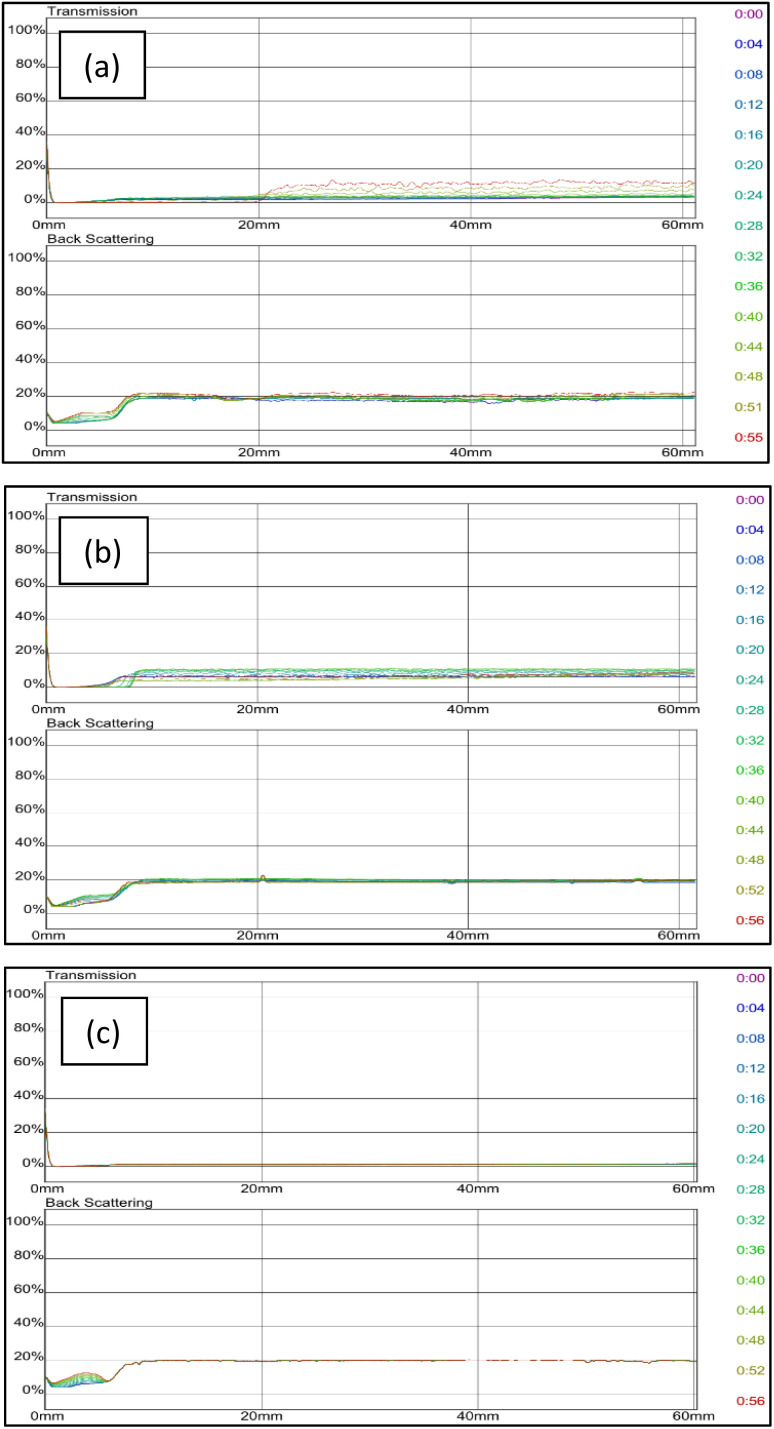
Transmission and backscattering profiles of grafted bentonite modified with zeolites with (a) Span 80 (b) Tween 80, and (c) combined Span 80 and Tween 80.

Δ*T* and ΔBS intensities of grafted bentonite modified with zeolites and coated with nonionic surfactant (Span 80 and Tween 80) are investigated. Fig. 13 shows the transmission (*T*) and backscattering (BS) intensities as a function of sample height over the analysis period for the dispersion of grafted bentonite modified with (a) zeolites and Span 80, (b) zeolites and Tween 80 and (c) zeolites and combined Span 80 and Tween 80.

Prior to the addition of non-ionic surfactants in graft copolymers zeolites, both transmittance and backscattering intensities at the bottom and upper of the bottle test fluctuate due to the uneven distribution of particles. Meanwhile, after the addition of either Span 80 or Tween 80 in the formulation, the transmitted light remained relatively stable. As shown in Fig. 13(a) and (b), the transmission intensities value of grafted bentonite modified with Span 80 and Tween 80 tended to be stable at 12% and 10% after 60 minutes, respectively. When the conformance formulation was modified with either Span 80 or Tween 80, the particles displayed slow sedimentation, as indicated by reduced transmittance at the vial's top and increased transmittance at the bottom. The particle sedimentation weakened the toughness and strength of the cross-linked gel. When the gel was compressed or stretched, the applied force was absorbed by the aggregates. The chemical bonds of acrylamide cross-linking and hydrogen bonding was stronger than the electrostatic and van der Waals forces holding the aggregates together. As a result, the aggregates were more easily torn apart, making the gel more prone to break and reduce its overall toughness and strength.^25^ Moreover, the stability is further improved by adding combined non-ionic surfactants, Span 80 and Tween 80, which have the best dispersion stability. In Fig. 13(c) or sample graft copolymer zeolites and combined Span 80 and Tween 80, the transmission remained constant at 1% after 60 minutes, indicating that the bentonite stayed uniformly dispersed throughout the sample. It's evidence that bentonite coated with zeolites and Span 80, and zeolites with Tween 80 showed reduced dispersion stability in contrast to zeolites with Span 80 and Tween 80. Hence, it seems that bentonite in the presence of zeolites and combined Span 80 and Tween 80 has good effect on bentonite particle dispersion stability. As reported by He *et al.*^45^ a nonionic surfactant exhibits excellent salt tolerance, a low critical micelle concentration, minimal sensitivity to strong acids and bases, high stability, and good compatibility with other surfactants. Nonionic surfactants also were used as a dispersant for silica nanoparticles, and they revealed that, the surfactants system exhibits a synergistic effect, improving its ability to dislodge oil droplets and alter wetting behavior.^29^ These findings are better than original grafted bentonite without modification with zeolites and non-ionic surfactants as in Fig. 11(c), which the transmission value is stable at 4% after 60 minutes.

**Fig. 14:**
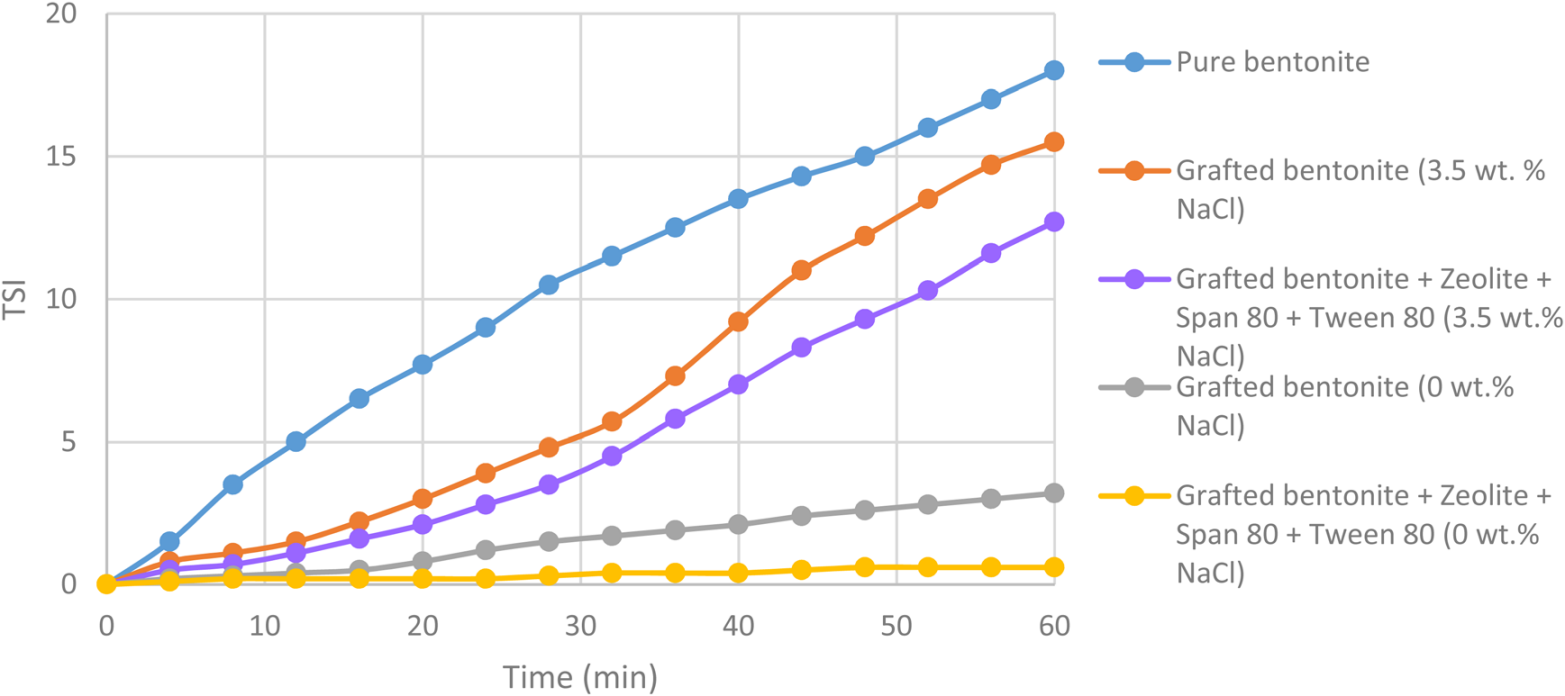
Turbiscan Stability Index (TSI) of pure bentonite, grafted bentonite and modified grafted bentonite.

Dispersion stability is a crucial indicator for grafted bentonite systems. High dispersion stability enhances the effective concentration retention of grafted bentonite during preparation, injection, and migration, thereby improving the plugging efficiency of the dispersed system. To further assess the sedimentation kinetics of the dispersions, the mean values of light transmission of both grafted bentonite and modified grafted bentonite have been compared over time. Fig. 14 illustrates the effect of pure bentonite, grafted bentonite and modified grafted bentonite on mean transmission value for the middle part of the samples (10–60 mm). The Turbiscan Stability Index (TSI) values increase over time, indicating growing instability in the dispersion. TSI values for grafted bentonite and modified grafted bentonite in 3.5 wt% NaCl shows higher instability in dispersion than in 0 wt% NaCl. Grafting introduces hydrophobic organic groups to bentonite, improving its compatibility with organic phases. However, saline conditions modify water structuring and polarity, thus affecting the solubility of the grafted polymers. As a result, phase separation may occur, leading to instability in the grafted bentonite layers. The dispersion stability of modified grafted bentonite in zeolites and combined Span 80 and Tween 80 was the strongest. The TSI value stabilized over 60 minutes below a value of 2, indicating that the stability level of the conformance control system was classified as ‘good’.^5,6^

### Rheology of RPM formulations

**Fig. 15:**
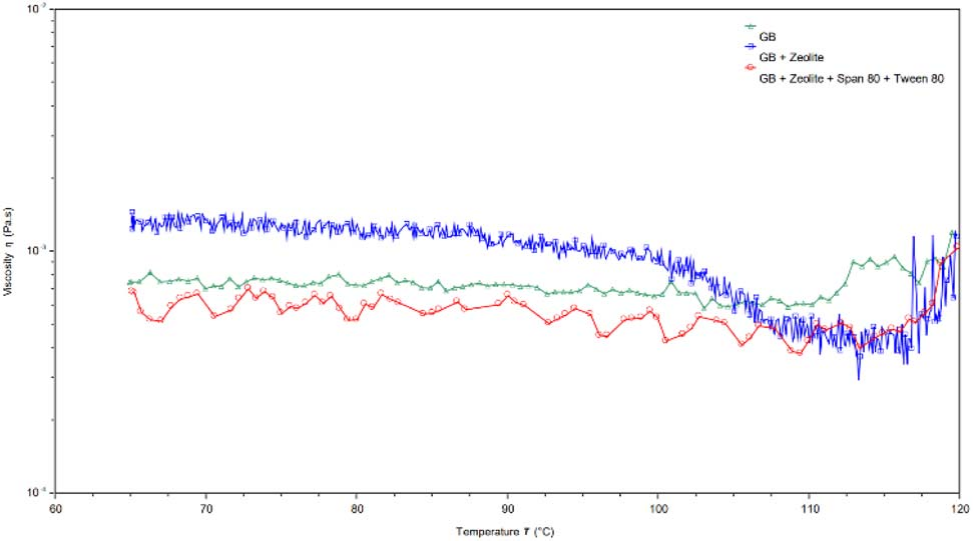
Effect of temperature on viscosity of GB and modified GB.

The viscosity of the RPM formulations is dependent on the temperature and the addition of zeolites and non-ionic surfactants (Span 80 and Tween 80). The results in Fig. 15 indicate that the viscosity of the dispersions slightly decreases as the temperature increases. However, at the transition temperature (∼110 °C), viscosity began to increase. This could be attributed to the disruption of particle interactions in clay suspensions as the temperature rises. Additionally, polyacrylamide (PAM) dispersions undergo thermally induced crosslinking, which contributes to a further increase in viscosity beyond a critical temperature.^46^ The viscosity of GB and zeolites exhibited greater variability compared to other dispersions, suggesting that the dispersions were less stable. Additionally, the viscosity was lower for GB and GB with zeolites and combined Span 80 and Tween 80, respectively indicating the successful synthesis of the dispersions. A lower viscosity dispersion is more suitable for conformance control.

### Particle size analysis (PSA)

**Fig. 16:**
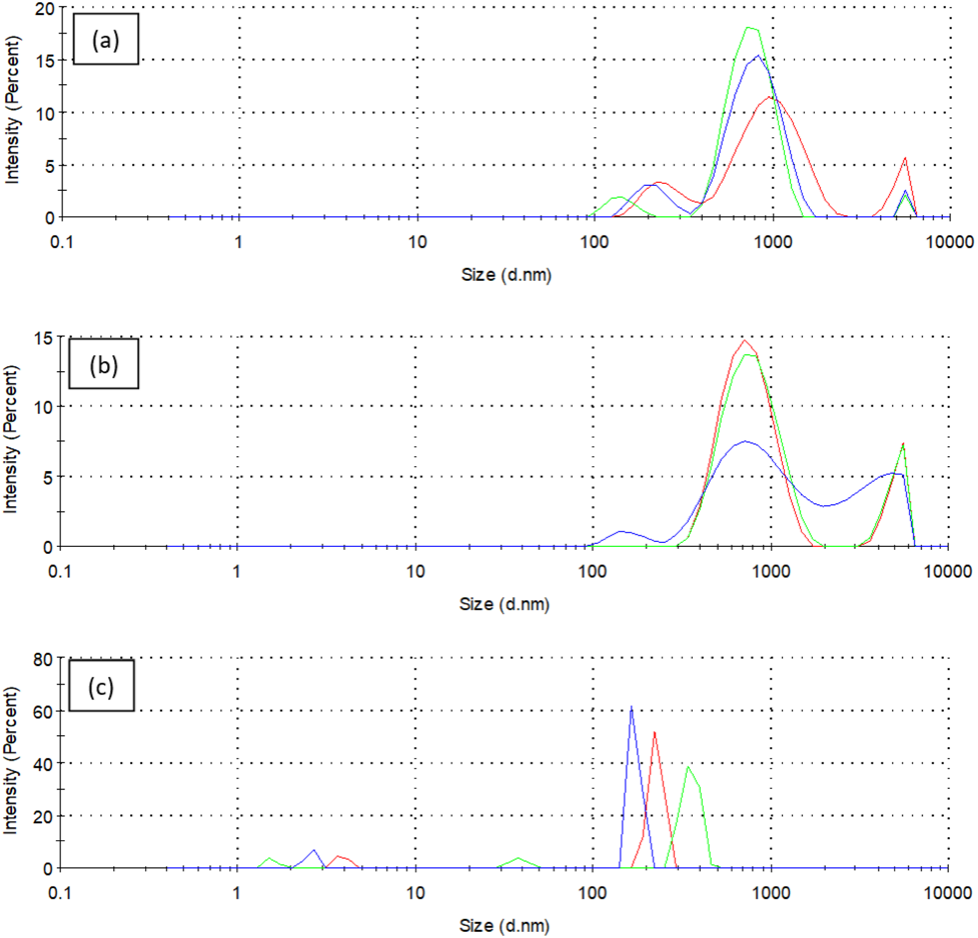
Particle size distribution for (a) grafted bentonite, (b) modified grafted bentonite with zeolites and (c) modified grafted bentonite with zeolites and non-ionic surfactants (Span 80 and Tween 80).

Particle size distribution plays a crucial role in determining the stability and performance of the modified bentonite dispersions. The analysis was conducted using Malvern Zetasizer Nano ZSP to assess changes in particle size before and after modification. The average particle size of grafted bentonite (GB) and GB with zeolites was 0.9 μm and 0.93 μm, respectively, while the modified grafted bentonite showed a size reduction of 0.3 μm, attributed to polymer intercalation. As illustrated in Fig. 16, the intensity curves for GB and modified GB with zeolites exhibit similar trends. The presence of a second peak at a larger size range indicates particle aggregation or secondary structure formation. The decrease in particle size following modification with Span 80 and Tween 80 suggests effective polymer intercalation within the bentonite layers. This observation aligns with previous studies, where polymer-functionalized clays demonstrated smaller hydrodynamic diameters, likely due to surface modification and enhanced steric stabilization.^40^

## Conclusions

This work was devoted to assess the colloidal dispersion stability of grafted bentonite as conformance control. This was performed by investigating clay and zeolite particles with crosslinked polymer gels. Grafted bentonite was modified at 5 conditions: (1) ratios of monomer, AM and AMPS, (2) salinities, (3) zeolites, (4) anionic surfactants, SDS, and (5) nonionic surfactants, Span 80 and Tween 80. Since grafted bentonite with polymer ratio of 42% : 58% of AM is the most stable compared to the two other polymers concentrations, it was chosen to be the optimum grafted bentonite to be modified in saline water, zeolites and surfactants. The results of multiple light scattering analysis indicate that bentonite in the presence of zeolites and combined Span 80 and Tween 80 shows the best dispersion stability on bentonite particle. However, treatment with zeolites alone did not give the best performance. The suspended particles undergo coagulation–flocculation because of the addition of solid zeolite nanoparticle flocculant. Effective stability helps to maintain the concentration of GB during preparation, injection, and migration, which improves the plugging ability of the dispersed GB system and reduces economic costs. Overall, the presented results show that grafted bentonite modified with zeolites and non-ionic surfactants was classified as having “good” characteristics for conformance control.

## Data availability

Data for this article was generated and supported by the data available at journal papers:

• Effect of Surfactants and Grafted Copolymer on Stability of Bentonite Particles Dispersion in Brine System [URL – https://api.semanticscholar.org/CorpusID:19395243],

• Effects of Synthesis Parameters on the Performance of Crosslinked Co-Polymers with Clays for Conformance Control [URL – https://doi.org/10.1016/j.ptlrs.2023.07.006],

• Graft Copolymerization of N Isopropylacrylamide and Acrylic Acid on Bentonite Colloids for In-Depth Fluid Diversion [URL – https://pubs.acs.org/doi/10.1021/acs.energyfuels.6b02507].

## Author contributions

All authors contributed to the study conception and design. The details of author contribution are as follows: conceptualization: Sofiah Atirah Raya, Ismail Mohd Saaid, Dzeti Farhah Mohshim, Ahmad Amirhilmi A Razak, methodology: Sofiah Atirah Raya, investigation: Sofiah Atirah Raya, data curation: Sofiah Atirah Raya, conceptualization: Sofiah Atirah Raya, writing – original draft: Sofiah Atirah Raya, writing – review & editing: all authors, supervision: Ismail Mohd Saaid, Dzeti Farhah Mohshim, funding acquisition: Ismail Mohd Saaid, Ahmad Amirhilmi A Razak.

## Conflicts of interest

There are no conflicts to declare.
